# The Relation Between Television Viewing Time and Reading Achievement in Elementary School Children: A Test of Substitution and Inhibition Hypotheses

**DOI:** 10.3389/fpsyg.2021.580763

**Published:** 2021-10-18

**Authors:** Wilfried Supper, Frédéric Guay, Denis Talbot

**Affiliations:** ^1^Département des fondements et pratiques en éducation, Faculté des sciences de l’éducation, Université Laval, Quebec, QC, Canada; ^2^Département de médecine sociale et préventive, Faculté de Médecine, Université Laval, Quebec, QC, Canada

**Keywords:** television viewing time, reading achievement, reading for leisure, intrinsic motivation, inattention

## Abstract

Research has focused on the relations between television (TV) viewing time and children’s reading achievement. Two hypotheses have been proposed to explain this relation. The substitution hypothesis proposes that TV viewing distracts students from activities that are important for their learning. The inhibition hypothesis proposes that watching television inhibits important affective/cognitive skills. In this study, we test both hypotheses by estimating the relation between TV viewing time and reading achievement. We use the frequency of students’ leisure reading and the frequency of interactions between students and their parents as potential mediators to test the substitution hypothesis, whereas for the inhibition one, we use students’ intrinsic motivation to read and their level of inattention. Data come from the Québec Longitudinal Study of Child Development (QLSCD). Designed by the *Institut de la statistique du Québec*, QLSCD covers a wide range of themes. The QLSCD is representative of children in Québec and contains 2223 participants who were followed from 0 to 21 years old. The four structural models tested are built as follows: the TV viewing time at 6 years old predicts the four mediating variables at 8 years old, which in turn predicts reading achievement at 10 years old. In addition, we have tested models’ gender invariance. Results indicate that TV viewing time is not directly or indirectly associated with reading achievement. Specifically, it is not associated with the mediating variables of child-parent interactions, intrinsic motivation, and inattention. However, the frequency of leisure reading is negatively associated with the time spent watching TV. This association is very small (−0.07) and has no indirect effect on reading achievement. Finally, results do not vary according to the gender of the participants. Our results are in line with those of previous studies in the field and cast some doubts on the potential negative effects of TV viewing time on reading achievement.

## Introduction

Television (TV) viewing time has been widely criticized for its negative influence on children’s learning to read (Winn, 1977; Postman, 1986; Ennemoser and Schneider, 2007). Major concerns are that time spent on watching TV replaces reading activities, reduces children’s interest for reading, lowers language skills, makes children intellectually lazy, inattentive, and inhibits their imagination (Himmelweit et al., 1958; Winn, 1977; Hornik, 1981; Postman, 1986; Popper et al., 1995; Ennemoser and Schneider, 2007; Desmurget, 2011). In addition, the important portion of time children devote to this leisure activity might be a cause of concern. Indeed, several studies reveal that, on average, children spend as much time watching TV, as they do performing classroom tasks (Paik, 2000; Desmurget, 2011). This makes TV watching one of the most frequent hobbies for a majority of children (Rideout, 2016). It is therefore important to identify the extent to which the time spent watching TV affects children’s reading achievement (RA). For more than 60 years, research has focused on this relation. However, very few studies have investigated the processes that explain why TV viewing has been associated with lower RA. Among studies testing mediators, results are divergent (Kostyrka-Allchorne et al., 2017; Supper et al., in-press article). Researchers have proposed several hypotheses to explain this divergence, including content type (Wright et al., 2001; Ennemoser and Schneider, 2007), cultural differences (Ennemoser and Schneider, 2007), differences in methodological quality between studies (Zavodny, 2006; Gentzkow and Shapiro, 2008; Munasib and Bhattacharya, 2010), the existence of moderators (Paik, 2000; Razel, 2001), and a non-linear relationship (Paik, 2000; Razel, 2001; Huang and Lee, 2010). However, studies that have focused on these potential confounding effects also show conflicting results (for research that specifically addresses this topic, see: Kostyrka-Allchorne et al., 2017; Supper et al., 2021). For example, cultural differences or content differences are aspects that we feel are secondary to consider in order to better understand this divergence in results. Indeed, most hypotheses on TV viewing time and RA are based on the video format of the TV and not on the type of content or messages likely to be broadcasted. Simply watching more TV is expected to predict a decrease in the amount of time children spend on educational activities such as reading, regardless of the type of program shown or the cultural practices between children in different countries. Based on past research, it is therefore difficult to draw clear conclusions about how TV viewing time is associated with RA.

### Hypotheses Explaining the Negative Effects of Television Watching Time on Reading Achievement

Researchers propose several processes to explain why TV watching time could be negatively associated with RA (Himmelweit et al., 1958; Schramm, 1961; Hornik, 1981; Beentjes and Van der Voort, 1988). These processes can be classified into the substitution and the inhibition hypotheses.

*The substitution hypothesis* posits that the time children spend watching TV replaces the time they spend on other activities more susceptible to enhance their RA, such as reading, writing, and doing schoolwork (Hornik, 1981). This hypothesis therefore assumes that the different activities that make up children’s schedules reflect a zero-sum game (Peaucelle, 1969). That is, an increase in the time spent on one type of activity, for example watching TV, inevitably decreases the time devoted to other types of activities favorable to RA. Among these educative activities, the frequencies of leisure reading and of non-educational interactions between children and their parents seem to be relevant mediating variables for testing the substitution hypothesis.

Leisure reading helps children to provide cognitive efforts when needed, to mobilize reading strategies learned in school, to discipline themselves, and to solve problems (Bergin, 1992). Thus, a decrease in the time that children spend on leisure reading will result in less time spent on cognitive, behavioral, and affective processes that enhance their RA. For this reason, we have chosen leisure reading as a mediating variable to test the substitution hypothesis. Some studies conclude that the time spent watching TV is associated with a decrease in leisure reading time (Koolstra et al., 1996; Shin, 2004; Ennemoser and Schneider, 2007), while other studies indicate that there is no association between these two variables (Himmelweit et al., 1958; Schramm, 1961; Ritchie et al., 1987; Anderson et al., 2001; Wright et al., 2001).

Furthermore, children who watch more TV could deprive themselves from the positive effects of spending time with their parents, such as better language development (Zimmerman et al., 2009) or support for their academic success (Guay et al., 2007). Indeed, TV watching time is associated with fewer parent-child interactions (Zimmerman et al., 2007). Television viewing time is also associated with a decrease in time spent on homeworks (Johnson et al., 2007), which are usually supervised by parents. However, some studies do not support these negative relationships (Schramm, 1961; Nakamuro et al., 2013).

*The inhibition hypothesis* posits that watching TV negatively affects RA by altering certain cognitive (mainly attention and concentration) or affective components (mainly valuing effort and interest in reading and school learning; Himmelweit et al., 1958; Beentjes and Van der Voort, 1988). The rationale underlying this hypothesis is that TV broadcasts content that does not require a sustained effort of understanding or concentration (Hornik, 1981). In addition, the images’ speed, the quality of the visual and sound effects and the abundant supply of channels make this content entertaining and very stimulating, which gives children pleasure from the very first minutes (Winn, 1977; Postman, 1986). Thus, immediate and easy access to an entertaining activity could lead children to develop a certain “mental laziness” (Himmelweit et al., 1958; Beentjes and Van der Voort, 1988) which would discourage them from mobilizing efforts or concentration to be interested in reading activities. Intrinsic motivation (IM) to read, that is reading for the sole interest or pleasure it provides (Ryan and Deci, 2000), could therefore be negatively affected by the number of hours children spend watching TV. It is well known that a decrease in IM to read is associated with a decrease in cognitive and emotional commitment to reading (Guthrie et al., 2012), in reading comprehension (Malanchini et al., 2017), in perceived reading skills and in RA (Morgan and Fuchs, 2007). For this reason, we have chosen IM to read as a mediating variable to test the inhibition hypothesis. In line with this, one study suggests that TV viewing is negatively associated with favorable attitudes toward reading (a concept similar to IM; Koolstra et al., 1996) or with reading motivation (Anderson et al., 2001), while another concludes that these variables are not significantly linked (Ennemoser and Schneider, 2007).

In addition, when asked to perform less interesting educational activities, children who watch a lot of TV may find it difficult to sustain their attention and, as a result, become more easily distracted (Beentjes and Van der Voort, 1988). An increase in inattention is associated with a decrease in RA (Rabiner et al., 2000). For this reason, we selected “attention” as an additional mediating variable to test the inhibition hypothesis. In this regard, the majority of studies indicate that watching TV is associated with an increase in inattention (Christakis et al., 2004; Mehmet-Radji, 2004; Acevedo-Polakovich et al., 2006; Johnson et al., 2007; Miller et al., 2007; Maass et al., 2010; Swing et al., 2010; Schmiedeler et al., 2014). However, an important number of studies have also found that there is no association between these variables (see Table 1).

**TABLE 1:** Studies on the association between TV viewing time and mediating variables.

Authors	Year	Mediator	Results
Tomopoulos et al.	2010	Cognitive skills	Negative association
Lonner et al.	1985	Cognitive skills	NS
Acevedo-Polakovich et al.	2006	Attention	Negative association
Cheng et al.	2010	Attention	Negative association
Christakis et al.	2004	Attention	Negative association
Maass et al.	2010	Attention	Negative association
Mehmet-Radji	2004	Attention	Negative association
Miller et al.	2007	Attention	Negative association
Schmiedeler et al.	2014	Attention	Negative association
Swing et al.	2010	Attention	Negative association
Johnson. Cohen et al.	2007	Attention	Negative association
Ansari and Crosnoe	2016	Attention	NS
Foster and Watkins	2010	Attention	NS
Landhuis et al.	2007	Attention	NS
Parkes et al.	2013	Attention	NS
Stevens et al.	2009	Attention	NS
Stevens et Mulsow	2006	Attention	NS
Zimmerman and Christakis	2007	Attention	NS
Koolstra et al.	1996	Attitude toward reading/Leisure reading	Negative association
Ennemoser and Schneider	2007	Attitude toward reading	NS
Johnson et al.	2007	Negative attitude toward school	Negative association
Huang & Lee	2010	Negative behavior at school	Negative association
Nakamuro et al.	2013	Negative behavior at school	NS
Parkes et al.	2013	Negative behavior at school	NS
Zimmerman et al.	2007	Language development	Negative association
Tomopoulos et al.	2010	Language development	Negative association
Barr et al.	2010	Language development	Negative association
Blankson et al.	2015	Language development	NS
Duch et al.	2013	Language development	Negative association
Schmidt et al.	2009	Language development	NS
Ruangdaraganon et al.	2009	Language development	Negative association
Pagani et al.	2013	Language development	Negative association
Linebarger et al.,	2005	Language development	Negative association
Bittman et al.	2011	Language development	NS
Johnson et al.	2007	Homework	Negative association
Nakamuro et al.	2013	Homework	NS
Koolstra et al.	1997	Mental effort	Negative association
Barr et al.	2010	Executive functions	Negative association
Hamer et al.	2010	Cognitive functions	Negative association
Ennemoser and Schneider	2007	Reading	Negative association
Anderson et al.	2001	Motivation	NS
Sharif et al.	2010	Search for sensation	Negative association
Blankson et al.	2015	Numeracy skills test	NS
Schmidt et al.	2009	Visual motor skills	NS

*NS = Not statistically significant at the 5% level.*

In conclusion, the four mediating variables we chose to test the substitution and inhibition hypothesis are leisure reading, parent-child interaction, IM to read, and inattention. These mediators were chosen because they seem to be the ones that best fit the propositions of these two hypotheses (Hornik, 1981; Beentjes and Van der Voort, 1988; Koolstra et al., 1996; Ennemoser and Schneider, 2007).

### Children’s Age

The vast majority of children start watching TV long before they reach the age when they learn to read. Therefore, by the time these children begin to learn to read at school, they have developed the habit of watching TV frequently and then have potentially developed cognitive and affective components that could hamper their willingness to engage in more demanding activities, such as reading (Hornik, 1981; Beentjes and Van der Voort, 1988; Ennemoser and Schneider, 2007). Thus, it is relevant to test the inhibition and substitution hypotheses early in children’s development.

### Children’s Gender as a Potential Moderator

Testing gender as a potential moderator is important for several reasons. First, boys watch TV for longer periods than girls (Sisson et al., 2012). They are exposed to more violent content and shows that portray stereotypical representations of masculinity (Coyne et al., 2014). These TV shows are thus more likely to provide the viewer with greater visual stimulation, and may thereby make boys more susceptible to inhibition effects. Second, the majority of children have female reader models (Morin, 2014) and boys more frequently report that reading is a female activity (Clark, 2012). This stereotypical view of reading could lead boys to value this activity less and thus to be less interested in reading (Morin, 2014). Third, boys read less frequently for leisure (Morin, 2014) and their book choices are more circumscribed around comic books (Morin, 2014). Boys therefore read more pictorial books, and choose more frequently reading formats that are close to TV content. For this reason, the type of reading that boys prefer may be more easily replaced by TV content. These gender differences may thus moderate the relationship between TV viewing time and RA. In light of the above, we expected the following: for boys, the relationships connecting TV viewing to the mediators and RA should be stronger than those observed for girls. In other words, boys would be more prone to the inhibition and substitution effects.

### Contributions of This Study

This study contributes to the existing literature in several ways. First, no study has tested the moderating role of gender in the “TV→mediators→RA” sequence. Second, studies that have tested the relationship between TV viewing time and some mediating variables show contrasting results. It is therefore difficult to determine whether watching TV is associated with less leisure reading or increased inattention. Yet, according to studies testing the relationship between TV viewing and RA (Hornik, 1981; Beentjes and Van der Voort, 1988), these two variables are among those that allow the inhibition and substitution hypotheses to be tested in the most stringent way. Third, few studies have tested substitution and inhibition hypotheses simultaneously, aside from the one conducted by Ennemoser and Schneider (2007). However, this study has important limitations. On the one hand, the results presented are unclear and they are divided between those indicating that TV viewing time is negatively associated with RA and those revealing that there is no association between these variables. On the other hand, this study contains a very limited number of participants. It is therefore important to test the substitution and inhibition hypothesis with a larger representative sample. Indeed, it was important to conduct such research, both socially and scientifically. It allows for a more precise estimation of how TV viewing time is related to RA and, therefore, for more appropriate recommendations and interventions. For example, if TV viewing time decreased leisure time spent reading, but did not inhibit cognitive abilities, parents could ensure that viewing time does not interfere with their children’s reading time. Conversely, if TV viewing time were not related to reading time, but was related to language development, then it would be important to recommend a more systematic reduction in TV viewing time, especially for younger children. Testing these two hypotheses together, therefore, provides a better understanding of the relationship between TV viewing time and RA. Fourth, longitudinal studies testing IM to read and the frequency of parent-to-child interactions as mediating mechanisms in the “TV→RA” relationship are scarce.

### Goals and Hypotheses

The goal of this study is to better understand the processes likely to mediate the relationship between TV viewing time and children’s RA. In order to test the inhibition and substitution hypotheses, we will explore if the time that 6-year-old children spend watching TV predicts the frequency of their leisure reading, the frequency of their interaction with their parents, their IM to read and the level of inattention at the age of 8. Additionally, we will test if, in turn, these four variables predict their RA at the age of 10. If the substitution hypothesis is supported, the TV viewing time at 6 years old will be negatively associated with the frequency of their leisure reading and/or the frequency of their interaction with the parent at 8 years old. If the inhibition hypothesis is corroborated, then TV viewing time at 6 years of age will be negatively associated with IM to read at 8 years of age and/or will be positively associated with inattention at 8 years of age. Finally, we posit that boys are more likely than girls to be affected by substitution and inhibition effects: at equal TV viewing time, we expect the substitution and inhibition effects will be more marked for boys than for girls.

## Materials and Methods

### Participants

The data came from the Québec Longitudinal Study of Child Development (QLSCD). The QLSCD targeted the population of children who were born in Québec between 1997 and 1998. However, children living on Indigenous reserves, regions of Nord-du-Québec, Cree territory and Inuit territory as well as children born prematurely (gestation less than 24 weeks) were excluded (Jetté and Des Groseilliers, 2000).

Québec longitudinal study of child development contained data concerning 2223 children aged between 5 and 6 months at the time of recruitment. This sample was made up of 48.8% of females. Since young children could not have answered the various measures themselves, the QLSCD has asked parents or legal guardians to complete the measures. Mother is the «Person Most Knowledgeable» (PMK) of children’s behaviors in 98.4% of cases. To obtain a better consistency, the QLSCD has encouraged the PMK to be the same respondent over time. In terms of education level, 44% of PMKs and 28% of fathers held a high school diploma or did not have a diploma, 29% of the PMK and 41% of fathers had a non-university post-secondary diploma and 26% of PMK and 30% of fathers had a university degree. Furthermore, 86.5% of mothers and 84.5% of fathers were Québec natives. Finally, French was the mother tongue for most of the participants’ mothers (81%), followed by English (9%) and other languages (10%).

The variables used to test our hypotheses were measured at the ages of 5 months, and 6, 7, 8, and 10 years old. In Table 2, we have indicated the measurement times during at which each variable was measured.

**TABLE 2:** Descriptive statistics.

	Girls		Boys		Total population			
	Mean	SD	Mean	SD	Mean	SD	Attrition	Age (years)
Parental practices	4.6	0.8	4.6	0.8	4.6	0.8	33.0%	6
Parental practices	4.3	0.8	4.4	0.9	4.4	0.9	34.8%	8
Inattention	1.3	0.4	1.5	0.5	1.4	0.5	47.6%	7
Inattention	1.3	0.4	1.5	0.5	1.4	0.5	49.7%	8
Talk about school activities a	6.9	0.4	6.9	0.5	6.9	0.5	31.5%	8
IQ	80.5	17.4	80.2	16.9	80.4	17.2	47.6%	6
Parental valorization of grades	3.5	0.6	3.5	0.6	3.5	0.6	31.7%	8
Leisure reading	5.3	2.0	4.5	2.2	4.9	2.2	42.7%	7
Leisure reading	4.3	1.0	3.8	1.3	4.1	1.1	43.2%	9
Motivation to read	4.3	0.8	4.0	1.0	4.1	0.9	34.4%	8
Motivation to read	4.3	0.8	4.0	1.0	4.1	0.9	34.4%	9
Reading score	3.7	1.3	3.4	1.4	3.5	1.3	42.1%	7
Reading score	3.6	1.2	3.2	1.3	3.4	1.3	57.2%	11
PMK diploma	2.7	1.1	2.7	1.0	2.7	1.1	0.1%	0.4
TV viewing time	1.8	0.9	1.9	0.8	1.8	0.8	33.0%	6
Gender	1	0	0	0	0.5	0.0	0.0%	0.4

*Motivation, parental practices and inattention variables are items means corresponding to these constructs.*

### Measures

#### The Average Daily Television Viewing Time

This variable was assessed by the PMK, when the child was 6 years old. The following questions were asked: “How much time does your child spend watching TV during the week?” and “How much time does your child spend watching TV during the weekend?” These two questions came from the Canadian Community Health Survey. This measure was similar to measures of TV viewing time used in other surveys (e.g., the National Longitudinal Survey of Children). The average TV viewing time per day for 6 years-old in the QLSCD was 1 h and 50 min. This amount is comparable to the average in other surveys (Zimmerman and Christakis, 2005; Rideout, 2016).

#### Academic Reading Achievement

This variable was assessed by teachers. In this study, we have selected scores when the children were 7 years old and when they were 10 years old. Scores on RA were strongly correlated with other measures of academic performance (see Forget-Dubois et al., 2007). The teacher has reported children’s RA by answering the following question: “How would you assess the child’s current academic success in reading?” The answers were given on a scale from 1 to 5: (1 = among the first in the class; 5 = among the last in the class). Students’ scores were assessed at the end of the school year.

#### The Frequency of Leisure Reading

This measure came from the PMK’s response to the question: “How often does your child enjoy reading?” This variable has been assessed when the child was 6 years old and when the child was 8 years old. Answers were rated on a scale from 1 to 7 (1 = rarely or never; 4 = a few times a month; 7 = every day). Previous studies showed that this measure of reading time for leisure was associated with children’s RA at 8 years old (Tétreault and Desrosiers, 2014; Nanhou et al., 2016; Torppa et al., 2020; Manu et al., 2021).

#### Frequency of Non-educational Interactions Between the Child and the Parent

This scale was filled by the PMK. For our study, we used the responses that were provided when the child was 6 years old and when the child was 8 years old. This scale was adapted from a subscale of the Parenting Practices Scale by Strayhorn and Weidman (1988; Boivin et al., 2000) which aimed to measure the frequency of supportive and encouraging behaviors from parent to child. This scale presented an acceptable level of validity and fidelity (Strayhorn and Weidman, 1988; Boivin et al., 2000; Verhoeven et al., 2010). It includes 10 items when the child was 6 years old and 5 items when the child was 8 years old. To measure the frequency of non-educational interactions between the child and the parent, we only kept three items per measurement time: “How often do you talk or play with the child?”; “How often do you do a special activity that she/he enjoys?”; “How often do you get involved in sports, hobbies or games?”. Answers were given on a scale from 1 to 5 (1 = never; 5 = several times a day). Other items on this scale were excluded for two reasons. First, the removed items mainly measured the quality of parenting practices rather than the frequency of parent-child interactions (i.e.,: “In the past 12 months, when you talked to the child about behavior, in what proportion of the time did you congratulate?”). However, our substitution hypothesis targeted the frequency of interactions and not their quality. The exclusion of items that did not measure the frequency of interactions therefore allowed us to be more consistent with our hypothesis. Second, the three items that were selected are identical between the 2 measurement times. The internal consistency of our three items was 0.61 when the child was 6 years old and 0.63 when the child was 8 years old (Cronbach’s alpha).

#### Intrinsic Motivation for School Reading

This measure came from a scale that was filled by the child at 7 and 8 years old. Items were from a subscale of the “Elementary School Motivation Scale” (Guay et al., 2010) whose aim was to measure different forms of school motivation in reading, writing and mathematics for a population of elementary school children. The content validity, construct validity, and internal consistency of the scale has been supported (Guay et al., 2010). The IM for school reading was made up of 3 items (“I like reading”; “Reading interests me a lot”; “I read even when I don’t have to”) for which the answers were given on a scale from 1 to 4 (1 = always no, sometimes no, sometimes yes, 4 = always yes). In QLSCD, Cronbach’s alpha was 0.68 when the child was 7 years old and 0.68 at 8 years old.

#### Children’s Symptoms of Inattention

This measure was filled out by the teacher when the child was 7 years old and when the child was 8 years old. The items on this scale came from the Ontario Child Health Study (OCHS; Cardin et al., 2011). This scale had a good level of validity (Boyle et al., 1993; Romano et al., 2006; Cardin et al., 2011) and has been used in various studies for its ability to predict school achievement (Pingault et al., 2013). This scale was composed of 5 items (“was unable to concentrate”; “could not maintain her/his attention for a long time”; “was easily distracted”; “had difficulty pursuing any activity”; “was inattentive”). Answers were given on a scale from 1 to 3 (1 = never or not true; sometimes/a little; 3 = often or very). For two measurement times, when the child was 7 and 8 years old, the Cronbach’s alpha was 0.88.

#### Control Variables

Several authors have highlighted the important influence that confounding variables have on the relationship between TV viewing time and RA (Ennemoser and Schneider, 2007; Munasib and Bhattacharya, 2010). More specifically, the intelligence quotient (IQ), the level of education of the parents and the parental involvement determine both the time spent watching TV and the RA (Ennemoser and Schneider, 2007). Consequently, we controlled in our models the following four variables: parents’ education level, child’s IQ, parents’ interest in their child’s education and parents’ valorization of good grades (Ennemoser and Schneider, 2007; Munasib and Bhattacharya, 2010).

Some authors also suggest that the time spent watching TV and the RA potentially have reciprocal relationships (Munasib and Bhattacharya, 2010). In order to minimize the influence of these biases, we controlled our mediating and dependent variables by taking into account initial scores on these variables.

In this study, we used four covariates to test our hypotheses. First, the highest level of education that the PMK has achieved was measured by a Likert scale from 1 to 4 (1 = no diploma; 4 = university degree). Second, the Peabody Picture Vocabulary Test was administered to the children when they were 6 years old (Dunn et al., 1993). This IQ test was strongly correlated with other measures of intelligence (Childers et al., 1994) and it was used in several studies that focus on RA (Salla et al., 2016). Scores could range between 1 and 120. Third, the frequency of PMK talking to the child about school activities was assessed by the following question: “How often do you talk to your child about school activities or work?” Responses were given on a scale from 1 to 4 (1 = daily; 4 = rarely). This variable was measured when the child was 7 years old. Fourth, the value of academic performance was the PMK’s response to the question: “How important is it to you that your child has good grades in school?” This variable was associated with the child’s RA (Tétreault and Desrosiers, 2014). The answer was given on a scale from 1 to 4 (1 = very important; 4 = not important) and it was measured when the child was 7 years old.

### Statistical Analysis

#### Missing Data

The QLSCD contained a significant number of missing data as shown in Table 2. We treated these missing data with the “full information maximum likelihood” (FIML) procedure of Mplus (Muthén and Muthén, 2012).

#### Structural Equation Models

Our statistical analyses were performed with Mplus software (Version 7.4; Muthén and Muthén, 2012) and the results presented are standardized. The four models have been tested with the Maximum Likelihood Robust (MLR) estimator. Only the model that included leisure reading as a mediating variable was fully saturated. For the other three models, which contained latent constructs (interaction with the parent, motivation and inattention), we have correlated the error terms of identical items appearing at several measurement times (Marsh and Hau, 1996). In addition, we have assessed whether these three models fitted the data adequately. To do this, we have selected three indices: the “Comparative Fit Index” (CFI), the “Tucker-Lewis Index” (TLI) and the “Root-Mean-Square Error of Approximation” (RMSEA). Our models were considered well adjusted if the CFI and TLI indices were greater than 0.90 and if the RMSEA was less than 0.08 (Hu and Bentler, 1999). We used the “indirect model” procedure to calculate the size of the total and indirect effects (Frazier et al., 2004) of the TV viewing time on the 10-year old RA.

#### Gender Invariance

In order to test our hypothesis regarding gender, we performed invariance analyses, which consisted in constraining certain parameters of our models to be equal between girls and boys. These analyses were composed of eight models (Caron, 2019) ranging from the unrestricted model (Table 3, line 1) to the most restrictive model (Table 4, line 8). Model 1 did not constrain any parameters across genders. In model 2, factor loadings were constrained to equality across genders. In model 3, factor loadings were constrained to equality across measurement times. Models 2 and 3 offered the possibility to verify if the participants understood items in the same way over time and if gender differences existed in items comprehension. Thereafter, we constrained various parameters including the intercepts (model 4), the residual errors (model 5), the correlated uniquenesses (model 6), the variances and covariances (model 7) as well as the paths (model 8). The comparisons among these models were made as follows: when the more restrictive model indicated a decrease of 0.01 in CFI and TLI, but an increase of 0.015 in the RMSEA compared to the less restrictive one (e.g., model 2 vs. model 1), the least restrictive model would be considered as better fitting the data.

**TABLE 3:** Fit indices for the 4 models with and without invariance test.

	Npar	χ^2^	*dl*	CFI	NNFI	RMSEA	[CI]
**Model with intrinsic motivation as a mediator**							
Model for the whole population	82	84.16*	38	0.98	0.96	0.02	[0.01, 0.03]
1-Configural model (sex)	142	78.73*	66	0.99	0.99	0.01	[0.00, 0.02]
2-Saturation (S) between sexes	138	81.30*	70	0.99	0.99	0.01	[0.00, 0.02]
3-(S) + saturation of identical items over time (ST)	136	82.84*	72	0.99	0.99	0.01	[0.00, 0.02]
4-(S) + (ST) + intercepts (I)	130	94.03*	78	0.99	0.99	0.01	[0.00, 0.02]
5-(S) + (ST) + (I) + residual errors (U)	124	192.03*	84	0.96	0.93	0.03	[0.03, 0.04]
5a-(S) + (ST) + (I) + residual errors (U) of item 2 and 3 are relaxed	128	103.87*	80	0.99	0.98	0.016	[0.01, 0.03]
6-(S) + (ST) + (I) + U + correlation of u of identical items over time (CU)	125	105.16*	83	0.99	0.99	0.016	[0.00, 0.02]
7-(S) + (ST) + (I) + U + (CU) + Var-cov (CV)	98	158.81*	110	0.98	0.97	0.02	[0.01, 0.03]
8-(S) + (ST) + (I) + U + (CU) + (CV) + Path	89	202.03*	119	0.97	0.96	0.03	[0.02, 0.03]
**Model with inattention as mediator**							
Model with the whole population	82	50.10*	37	0.99	0.99	0.01	[0.00, 0.02]
1-Configural model (sex)	142	77.05*	66	0.99	0.99	0.01	[0.00, 0.02]
2-Saturation (S) between sexes	138	84.81*	70	0.99	0.99	0.01	[0.00, 0.02]
3-(S) + saturation of identical items over time (ST)	136	85.82*	72	0.99	0.99	0.01	[0.00, 0.02]
4-(S) + (ST) + intercepts (I)	130	90.09*	78	0.99	0.99	0.01	[0.00, 0.02]
5- (S) + (ST) + (I) + residual errors (U)	124	118.47*	84	0.99	0.99	0.02	[0.01, 0.03]
6-(S) + (ST) + (I) + U + correlation of u of identical items over time (CU)	121	118.94*	87	0.99	0.99	0.03	[0.01, 0.03]
7-(S) + (ST) + (I) + U + (CU) + Var-cov (CV)	94	176.76*	114	0.99	0.99	0.03	[0.02, 0.03]
8-(S) + (ST) + (I) + U + (CU) + (CV) + Path	85	234.15*	123	0.98	0.98	0.03	[0.02, 0.03]
**Model with frequency of reading as mediator**							
Model with the whole population	82	67.49*	37	0.98	0.96	0.02	[0.01, 0.03]
1-Configural model (sex)	142	104.27*	66	0.98	0.96	0.02	[0.01, 0.03]
2-Saturation (S) between sexes	138	112.20*	70	0.98	0.95	0.02	[0.02, 0.03]
3-(S) + saturation of identical items over time (ST)	136	117.47*	72	0.98	0.95	0.02	[0.02, 0.03]
4-(S) + (ST) + intercepts (I)	130	121.77*	78	0.98	0.96	0.02	[0.02, 0.03]
5- (S) + (ST) + (I) + residual errors (U)	124	126.39*	84	0.98	0.96	0.02	[0.01, 0.03]
6-(S) + (ST) + (I) + U + correlation of u of identical items over time (CU)	121	128.31*	87	0.98	0.96	0.02	[0.01, 0.03]
7-(S) + (ST) + (I) + U + (CU) + Var-cov (CV)	94	167.44*	114	0.98	0.96	0.02	[0.01, 0.03]
8-(S) + (ST) + (I) + U + (CU) + (CV) + Path	85	172.79*	123	0.97	0.96	0.02	[0.01, 0.03]
**Model with leisure reading as mediator**							
Model with the whole population	65	0.00*	0	1.00	1.00	0.00	[0.00, 0.00]
1-Configural model (sex)	108	0.00*	0	1.00	1.00	0.00	[0.00, 0.00]
2-Var-cov (CV)	81	44.52*	27	0.98	0.95	0.02	[0.01, 0.04]
3-(CV) + Path	72	58.82*	36	0.97	0.95	0.02	[0.01, 0.04]

Npar is the number of parameters estimated; dl is the degree of freedom; CFI is the “Comparative Fit Index”; TLI is the “Tucker-Lewis Index” and RMSEA is the “Root Mean Square Error of Approximation.” *Means that statistically significant at the 5% level.

**TABLE 4:** Correlations between not answering one of these variables with gender and the PMK diploma (0 = answer provided and 1 = no answer).

	Gender	PMK diploma
Parent-child interaction item 1 to 6 years old	–0.06	–0.10
Parent-child interaction item 2 to 6 years old	–0.07	–0.12
Parent-child interaction item 3 to 6 years old	–0.06	–0.10
Parent-child interaction item 1 to 8 years old	–0.07	–0.12
Parent-child interaction item 2 to 8 years old	–0.06	–0.10
Parent-child interaction item 3 to 8 years old	–0.07	–0.12
Inattention item 1 to 7 years old	–0.09	–0.08
Inattention item 2 to 7 years old	–0.10	–0.06
Inattention item 3 to 7 years old	–0.09	–0.08
Inattention item 1 to 8 years old	–0.10	–0.06
Inattention item 2 to 8 years old	–0.09	–0.08
Inattention item 3 to 8 years old	–0.10	–0.05
Leisure reading at 6 years old	–0.09	–0.15
Leisure reading at 8 years old	–0.07	–0.10
Intrinsic motivation item 1 to 7 years old	–0.10	–0.09
Intrinsic motivation item 2 to 7 years old	–0.10	–0.07
Intrinsic motivation item 3 to 7 years old	–0.10	–0.09
Intrinsic motivation item 1 to 8 years old	–0.10	–0.07
Intrinsic motivation item 2 to 8 years old	–0.10	–0.09
Intrinsic motivation item 3 to 8 years old	–0.10	–0.07
Reading achievement at 7 years old	–0.09	–0.07
Reading achievement at 10 years old	–0.06	–0.09
TV viewing time at 6 years old	–0.06	–0.09

*All correlation coefficients are statistically significant at p < 0.05.*

## Results

### Preliminary Analyses

#### Missing Data

According to Koolstra et al. (1996, 1997), missing data are associated with lesser RA and higher amount of TV viewing. Thus, it was important to adjust for missing data to avoid bias. Among all of our variables, the gender and diploma of the PMK showed little missing data (see Table 2). These two variables allowed us to compare the participants in our sample who had missing data with those who did not. To compare these two subsamples, we first computed a dichotomized score based on responses provided on TV viewing time, RA, IM, leisure reading, inattention and parent-child interaction. Those who had non-missing scores on these variables were assigned 0, whereas those who had missing scores were assigned 1. Thus, six dummy variables were computed. Then, we correlated these six dummies to the gender and educational degree of the PMK. Results (see Table 4) indicate that more missing values are observed among boys, r between –0.052 and –0.104 and children who live in a household where the PMK has a low level of education, r between –0.06 and –0.12. However, these correlations were quite low.

Thus, the estimation of our models was carried out using the FIML procedure (Muthén and Muthén, 2012), which is a more robust procedure than complete case analysis or imputation with the mean (Caron, 2019). In addition, several participants (29.8%, *n* = 644) presented missing data on more than one variable included in our models. We thus tested our models with and without these participants and we did not observe any meaningful difference in the results. For this reason, all participants were kept in our analyses.

#### Descriptive Statistics

Table 5 indicates that TV viewing time is statistically significantly associated with RA at 7 and 10 years old, as well as inattention, leisure reading frequency, and the frequency of parent-child interactions at 6 years old. Although statistically significant, all of these relationships were nonetheless very modest. Table 2 shows that, at all measurement times, boys read less frequently for leisure than girls, that they have on average a lower level of IM to read as well as a lower RA. However, there were no gender differences in the average time spent watching TV at 6 years old.

**TABLE 5:** Correlations between variables of the 4 models.

	Variables	1	2	3	4	5	6	7	8	9	10	11	12	13	14	15
1	TV viewing time at 6 years old	–														
2	Inattention at 7 years old	0.08*	–													
3	Inattention at 8 years old	0.10*	0.66*	–												
4	Motivation to read at 7 years old	–0.05	−0.25*	−0.19*	–											
5	Motivation to read at 8 years old	–0.05	−0.2*	−0.18*	0.37	–										
6	Parental practices at 6 years old	−0.14*	–0.03	0.01	–0.02	0.046	–									
7	Parental practices at 7 years old	–0.04	−0.10*	–0.04	0.10*	0.08*	0.61*	–								
8	Gender	–0.03	−0.22*	−0.22*	0.16*	0.18*	–0.03	–0.02	–							
9	PMK diploma	−0.18*	−0.22*	−0.21*	0.08*	0.04	0.10*	0.07	–0.00	–						
10	IQ at 6 years old	−0.08*	−0.30*	−0.29*	0.03	0.12*	0.08*	0.13*	0.019	0.24*	–					
11	Talk about school activities 7 years old	–0.03	0.00	–0.01	0.01	0.10*	0.12*	0.18*	0.03	0.08*	0.03	–				
12	parental valorization of grades	0.02	–0.05	–0.03	0.05	0.12*	–0.05	–0.03	0.02	−0.07*	−0.08*	0.027	–			
13	Reading score at 7 years old	−0.13*	−0.57*	−0.49*	0.27*	0.22*	0.07	0.04	0.12*	0.28*	0.40*	0.03	0.07*	–		
14	Reading score at 10 years old	−0.09*	−0.49*	−0.53*	0.16*	0.23*	0.03	0.09*	0.18*	0.24*	0.42*	0.02	0.05	0.62*	–	
15	Leisure reading at 6 years old	−0.08*	−0.16*	−0.07*	0.02*	0.12*	0.14*	0.11*	0.18*	0.07*	0.08*	0.07*	0.05	0.14*	0.14*	−
16	Leisure reading at 8 years old	−0.11*	−0.18*	−0.15*	0.22*	0.35*	0.13*	0.12*	0.23*	0.07*	0.10*	0.11*	0.05	0.23*	0.22*	0.26*

The * indicates a p-value less than 5% Motivation, parental practices and inattention variables are latent constructs.

### Models Tested

#### Hypothesis Testing

Table 3 shows the results of the fit indices for the four structural equation models tested. These indices show a very good level of fit because the CFI and TLI values are above 0.95 and the RMSEA values are below 0.025.

Table 6 presents the beta and the standard error of paths from the four models. Among the covariates, IQ at 6 years old and PMK diploma are the most associated with the mediator variables and the RA variables. In addition, the PMK diploma is the most important predictor of TV viewing time. These results corroborate those of other studies (Koolstra et al., 1997; Ennemoser and Schneider, 2007).

**TABLE 6:** All beta and standard error values for the four models (including parameters for covariates).

	Mediator at 8 years old		Reading achievement at 7 years old		Reading achievement at 10 years old		TV viewing time at 6 years old		Mediator at 7 years old	
					
Model with inattention as mediator	Beta	SE	Beta	SE	Beta	SE	Beta	SE	Beta	SE
TV viewing time at 6 years old	0.03	0.03	–0.06	0.03	0.01	0.03			0.03	0.03
Inattention at 7 years old	0.55*	0.03			–0.03	0.05				
Inattention at 8 years old					−0.25*	0.04				
Gender^a^	−0.11*	0.03	0.03	0.02	0.07*	0.03	–0.03	0.03		
PMK diploma	–0.03	0.03	0.19*	0.03	0.03	0.03	−0.18*	0.02	−0.15*	0.03
IQ at 6 years old	–0.04	0.03	0.34*	0.03	0.19*	0.03			−0.25*	0.03
Talk about school activities at 7 years old	0.01	0.03			–0.01	0.03				
parental valorization of grades at 7 years old	–0.00	0.03			0.03	0.03				
Reading achievement at 7 years old	−0.15*	0.04			0.38*	0.03				
**Model with intrinsic motivation as mediator**										
TV viewing time at 6 years old	–0.02	0.03	−0.07*	0.03	0.00	0.03			–0.04	0.03
Intrinsic motivation at 7 years old	0.32*	0.04			–0.04	0.04				
Intrinsic motivation at 8 years old					0.09*	0.03				
Gender	0.13*	0.03	0.09*	0.03	0.10*	0.03	–0.03	0.03		
PMK diploma	–0.03	0.03	0.19*	0.03	0.05	0.03	−0.18*	0.02	0.07*	0.03
IQ at 6 years old	0.08*	0.04	0.34*	0.03	0.20*	0.03			0.02	0.03
Talk about school activities 7 years old	0.09*	0.04			–0.02	0.03				
parental valorization of grades at 7 years old	0.09*	0.03			0.03	0.03				
Reading achievement at 7 years old	0.09*	0.04			0.50*	0.03				
**Model with frequency of PMK-child interactions as mediator**										
TV viewing time at 6 years old	0.04	0.04	−0.06*	0.03	–0.01	0.03				
PMK-child interactions at 6 years old	0.60*	0.05	0.01	0.04	–0.09	0.05				
PMK-child interactions at 8 years old					0.09	0.05				
Gender	0.00	0.03	0.12*	0.07	0.11*	0.03	–0.03	0.03	–0.03	0.04
PMK diploma	–0.01	0.04	0.19*	0.03	0.05	0.03	−0.18*	0.02	0.10*	0.04
IQ at 6 years old	0.10*	0.04	0.34*	0.03	0.20*	0.03				
Talk about school activities 7 years old	0.11*	0.04			–0.01	0.03				
parental valorization of grades at 7 years old	0.01	0.03			0.03	0.03				
Reading achievement at 7 years old	–0.03	0.04			0.51*	0.03				
**Model with leisure reading as mediator**										
TV viewing time at 6 years old	−0.07*	0.03	–0.05	0.03	0.07	0.03				
Leisure reading at 6 years old	0.19*	0.03	0.09*	0.03	0.01	0.03				
Leisure reading at 8 years old					0.07*	0.03				
Gender	0.17*	0.03	0.10*	0.03	0.09*	0.03	–0.03	0.03	0.18*	0.03
PMK diploma	–0.00	0.03	0.19*	0.03	0.05	0.03	−0.18*	0.02	0.06*	0.03
IQ at 6 years old	0.01	0.03	0.33*	0.03	0.21*	0.03				
Talk about school activities 7 years old	0.07*	0.03			–0.02	0.03				
parental valorization of grades at 7 years old	0.03	0.03			0.03	0.03				
Reading achievement at 7 years old	0.16*	0.04			0.49*	0.03				

*indicates a p-value less than 5%.

*^a^0 = boy and 1 = girl.*

**FIGURE 1:**
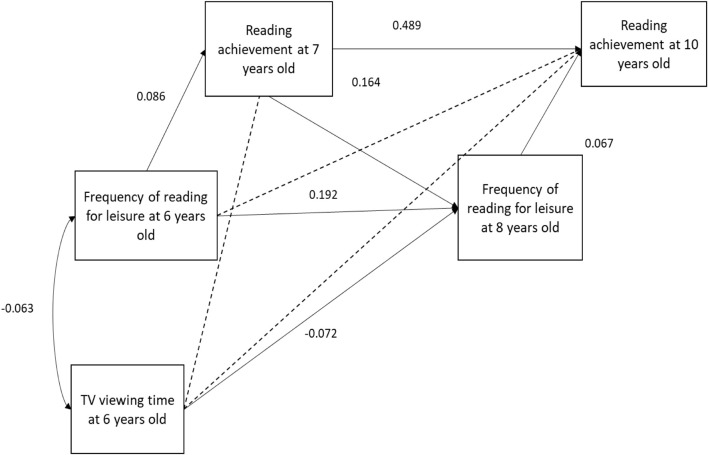
Leisure reading mediation model. Dotted line indicates a *p*-value above 5%.

**FIGURE 2:**
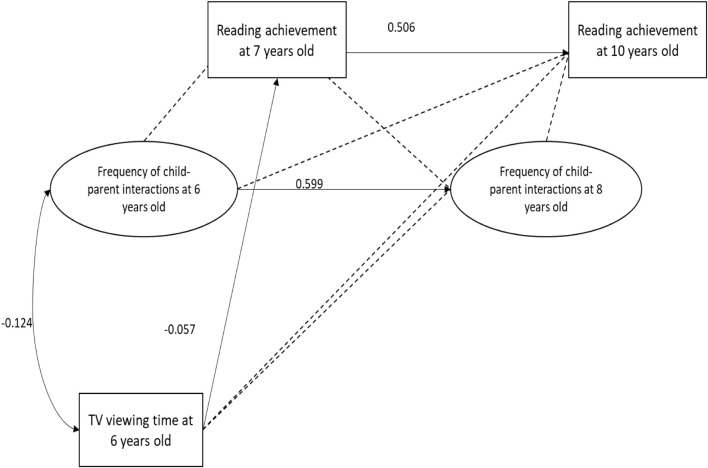
Parent-child interaction mediation model. Dotted line indicates a *p*-value above 5%.

**FIGURE 3:**
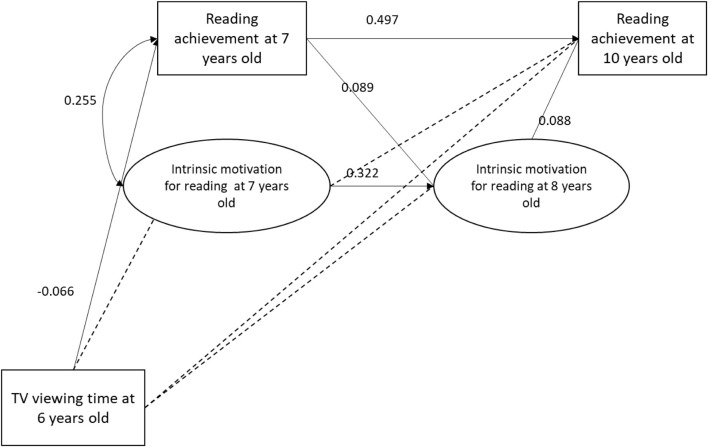
Intrinsic motivation mediation model. Dotted line indicates a *p*-value above 5%.

**FIGURE 4:**
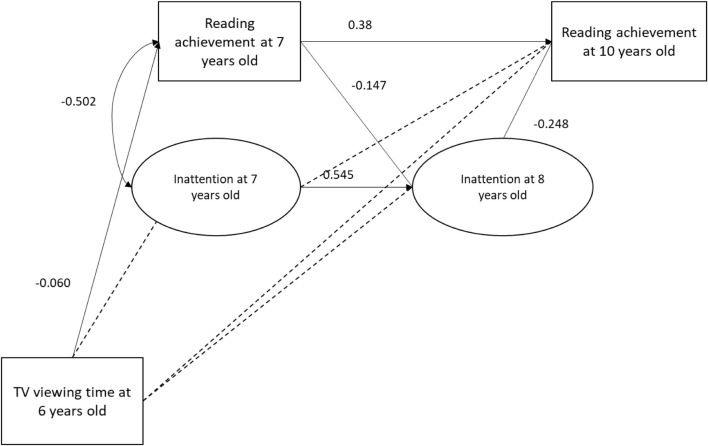
Inattention mediation model. Dotted line indicates a *p*-value above 5%.

Figures 1–4 present the results of the four models that test the substitution (Figures 1, 2) and inhibition (Figures 3, 4) hypotheses. Among these four models, the only mediator to be associated with TV viewing time was the frequency of leisure reading. More specifically, TV viewing time at 6 years old was negatively associated to leisure reading frequency at 8 years old (β = −0.072; SE = 0.033). However, this association was too small to produce indirect effects on RA at 10 years old (β = −0.005; *p* = 0.117).

Table 7, which presents the results of the indirect and total effects, indicates that, with the exception of the parent-child interactions model, the indirect effects of our models are statistically significant, but with small effect sizes (β between −0.036 and −0.032). First, these indirect effects of TV viewing time at 6 years old on RA at 10 years old were caused almost exclusively by the link between TV viewing time and RA at 7 years old. Because these indirect links were not caused by the mediating variables from which we have tested the substitution or inhibition effects, our results therefore did not support the inhibition and substitution hypotheses. Second, there was no statistically significant total effect in the 4 models. This result means that the sum of the direct and indirect effects of the 6 year olds TV viewing time toward the 10 years old RA was not statistically different from zero.

**TABLE 7:** Indirect and total effects.

Model	Total effect	*p*-value	Indirect effect	*p*-value
Reading	−0.03	0.41	−0.03	0.03
Parental practices (PP)	−0.03	0.00	−0.03	0.10
Motivation	−0.03	0.29	−0.03	0.02
Inattention	−0.03	0.34	−0.04	0.03

#### Invariance Analysis

The results of our invariance analysis indicated that there were no differences between the two groups concerning the associations between the time spent watching TV, our 4 mediators and RA. First, the results of models that tested parent-child attention and interaction as mediators yielded poorer model fit indices (see Table 3). Second, in the model where the IM is the mediator, our results indicate a drop in the acceptable level of the adjustment indices when residual errors are constrained (Table 3, A a q line 5). We therefore removed some of these constraints (see model 5a) and we then constrained the correlated uniquenesses (model 6), the variances and covariances (model 7) as well as the paths (model 8). For model 8, results indicate a little drop in CFI and NNFI values. Thus, we relaxed these constraints and we calculated the differences between genders for the regression coefficients. Our results show small differences between gender for association between IM at 7 years old and motivation at 8 years old, and between TV viewing time at 6 years old and RA at 7 years old. However, there is no difference between genders for associations between TV viewing time at 6 years old and IM at 8 years old and between the TV viewing time at 6 years old and RA at 10 years old (Table 8). Third, in the model where leisure reading is the mediator, our results indicate a decrease in the acceptable level of adjustment indices for the Residual Invariance Model (Table 3, model 2). However, our results do not indicate a further decline in these adjustment indices when the path coefficients are constrained to equality (Table 3, model 7). Thus, in this model with leisure reading as a mediator, the differences between genders were only found on the variances/covariances and not on the relationship between the time spent watching TV, leisure reading, and RA. In sum, the invariance analysis performed on the four models did not corroborate our second hypothesis, which proposed that boys are more exposed than girls to the effects of substitution and inhibition.

**TABLE 8:** Results of gender differences.

	β	Standard error	*p*-value
Intrinsic motivation at 8 years old	0.06	0.05	0.22
RA at 10 years old	−0.07	0.06	0.23

## Discussion

The goal of this study was to estimate the contribution of four potential mediating variables (leisure reading, child-parent interaction frequency, IM to read and the level of inattention) explaining the relationship between TV viewing time and RA as a function of gender. These four mediators were chosen to test the substitution and inhibition hypotheses. In addition, we hypothesized that the size of the substitution and inhibition effects would be greater for boys. Overall, our results did not support these assumptions. First, only the leisure reading frequency was negatively associated with TV viewing time at 6 years old, however, this negative association was very small and had no indirect effect on RA at 10 years old. Second, relationships between TV viewing time at 6 years old and our mediating variables at age 9 and RA at age 10 did not vary across genders. Therefore, our results do not corroborate the substitution and inhibition hypotheses, nor do they corroborate our hypothesis proposing that TV viewing would be more detrimental to boys’ RA than to the one of girls.

The substitution hypothesis assumes that the time children spend on activities that are favorable to their RA and the time they spend watching TV is organized as a zero-sum game (Peaucelle, 1969). However, our results show, that activities that are favorable to RA are not, or only slightly, replaced by the time that students spend watching TV. One explanation for these results is that children watch TV during times when they probably would not have chosen to perform activities more favorable to their RA. Furthermore, our results also suggest that children practice activities favorable to their RA when they are not permitted to watch TV. For example, children who read at night before sleep when their parents make sure that they cannot watch TV would not see time they spend on this activity decrease if they are more exposed to TV in afternoon. Thus, children who watch more TV are not doing this activity at the expense of the time they spend leisure reading or interacting with their parents.

The inhibition hypothesis is also not corroborated. Specifically, results indicate that there is no association between TV viewing time and IM to read and inattention. A first explanation could be that principles on which the inhibition hypothesis is based are wrong. Specifically, it may be inadequate to propose that TV viewing is a leisure that does not require effort and attention and thus could induce in children “mental laziness”. A second possibility is that the negative influence of TV is not important enough to translate into a measurable drop in IM to read and to an increase in inattention symptoms.

Gender moderation analyses did not indicate a difference between the two groups, which does not support our hypothesis. This hypothesis is based on the fact that boys and girls have a relationship with reading and TV that differs quantitatively and qualitatively. Our results indicate that, on the contrary, gender does not affect the relationship between TV viewing time, the mediating variables and the RA. As we have suggested, there may be substitution and inhibition moderators that have not been studied yet, such as social and economic status, age of children or the type of content children are watching. In this sense, if gender is not the most relevant moderator, the choice of another moderator should be considered in future studies.

## Limits

A first limitation of our study concerns the TV viewing time measure. Indeed, several researchers questioned the accuracy and validity of this measure particularly with regard to the level of measurement error it contains and its relation with social desirability (see Bryant et al., 2007 for a systematic review of these studies from 1985 to 2006; Atkin et al., 2012; Cabanas-Sánchez et al., 2018; Byrne et al., 2021). However, the impact of this shortcoming seems to be trivial for several reasons. Indeed, since the 1980s, the amount of TV viewing time obtained via a self-report measure has been compared to the time derived from objective measures (video or direct observation; see Bryant et al., 2007 for a systematic review of these studies from 1985 to 2006; Clark et al., 2009; Atkin et al., 2012; Cabanas-Sánchez et al., 2018; Aunger and Wagnild, 2020; Byrne et al., 2021). Researchers conclude that self-report questionnaires have an acceptable level of validity (Gorin et al., 2006; Otten et al., 2010; Dwyer et al., 2011; Foley et al., 2012; Wijndaele et al., 2014; Cabanas-Sánchez et al., 2018; Aunger and Wagnild, 2020; De Moraes et al., 2020). Second, the QLSCD comprised a social desirability scale that we used to calculate the correlation with the TV viewing time variable. This correlation is -0.04. Thus, TV viewing time does not seem to be affected by the degree of social desirability of the participants. Third, Munasib and Bhattacharya (2010) and Nakamuro et al. (2013) measured the impact that measurement error can have on the estimation of the relationship between TV viewing time and RA. These authors concluded that measurement error has no impact on results. For these reasons, although it is important to consider that the questionnaire has limitations in measuring TV viewing time, this aspect does not seem to invalidate our results.

Third, the mediating and dependent variables associated with the TV variable are spaced by an interval of two years between each time point. This time interval is imposed by QLSCD sampling. However, it is unknown if and how the duration of time between TV viewing, mediating variables and RA affects effect sizes. To our knowledge, this question of temporality on the link between TV and RA has not yet been studied. Thus, it would be relevant to address this question in further studies.

Fourth, if we have taken into account several important confounding variables, other sources of bias might nevertheless operate. A potential source of bias could come from parents who use TV as a means of reward and punishment (Huang and Lee, 2010). Indeed, this practice consists of increasing the time spent watching TV when children have good grades and decreasing it when children have poor ones. Such contingent use of TV would result in a positive association between TV and RA, that is not mainly attributable to the real effects of TV. However, it seems to us that this risk of bias is relatively low since our TV variable measures the viewing time of children before they enter primary school. Thus, the children in our sample are not subject to a school evaluation that parents could use to regulate their time spent watching TV.

Finally, TV viewing time was measured with 6 years old children. Our results are therefore limited to young children and do not seem to be replicable to an older population such as adolescents. In this regard, no study has tested the substitution and inhibition hypotheses jointly in a population of adolescents. It would therefore be interesting to test these two hypotheses with this population.

## Conclusion

The main concerns and criticisms linked to TV viewing are that it replaces reading in children’s leisure time, reduces their interest in this activity and increases their inattention, which would harm the development of their competencies at school. However, our results indicate that watching TV is not associated with lower RA and that the drop in the amount of time spent leisure reading is not enough to affect RA. On the social level, our results therefore provide useful input to the debate on TV. Our results do not support the substitution and inhibition hypotheses while controlling for important covariates. However, it seems wrong to consider that these results completely invalidate these two hypotheses for three reasons.

First, the research that has tested these two hypotheses presents mixed results. If some studies obtained results similar to ours, indicating that the time spent watching TV is very weakly and negatively associated with the time spent leisure reading (Koolstra et al., 1996; Ennemoser and Schneider, 2007) and that it is not associated with IM to read and inattention (Ansari and Crosnoe, 2016), other studies have shown different results (Ritchie et al., 1987; Koolstra et al., 1996; Anderson et al., 2001; Wright et al., 2001; Zimmerman et al., 2007). Our results should therefore be interpreted with caution.

Second, there is an increasing presence of new types of screens such as digital tablets, telephones or laptops (Kostyrka-Allchorne et al., 2017). These screens present major differences when compared to TV. Unlike a fixed screen, they allow the viewers to access a large variety of content easily and quickly, right in the palm of their hands, anywhere, anytime. Thus, it seems important to test the substitution and inhibition hypotheses in this context of new screens. Considering that watching TV shows is one of the main activities that children perform with these screens (Rideout, 2016) and considering that there is still little research on our subject, it therefore seems socially and scientifically important to emphasize the need to undertake additional studies in order to have a more substantiated knowledge on the relationship between exposure to TV or streaming programs, children’s RA and the mediators and moderators likely to explain that relation.

## Data Availability Statement

Publicly available datasets were analyzed in this study. This data can be found here: https://www.jesuisjeserai.stat.gouv.qc.ca/default_an.htm.

## Author Contributions

WS conducted the study process, performed data analysis, and drafted the manuscript. FG supervised the design of the study. FG and DT contributed to the data analysis and results interpretation, critically revised the article, and approved this version. All authors provided important contributions to the work.

## Conflict of Interest

The authors declare that the research was conducted in the absence of any commercial or financial relationships that could be construed as a potential conflict of interest.

## Publisher’s Note

All claims expressed in this article are solely those of the authors and do not necessarily represent those of their affiliated organizations, or those of the publisher, the editors and the reviewers. Any product that may be evaluated in this article, or claim that may be made by its manufacturer, is not guaranteed or endorsed by the publisher.
